# The relationship between body mass index before pregnancy and the amount of weight that should be gained during pregnancy: A cross-sectional study

**DOI:** 10.12669/pjms.35.5.133

**Published:** 2019

**Authors:** Resmiye Ozdilek, Yilda Arzu Aba, Sena Dilek Aksoy, Bulat Aytek Sik, Yasam Kemal Akpak

**Affiliations:** 1 Resmiye Ozdilek, Department of Midwifery, Faculty of Health Science, Kocaeli University, Izmit, Turkey; 2 Yilda Arzu Aba, Department of Nursing, Faculty of Health Science, Bandirma Onyedi Eylül University, Balikesir, Turkey; 3 Sena Dilek Aksoy, Department of Midwifery, Faculty of Health Science, Kocaeli University, Izmit, Turkey; 4 Bulat Aytek Sik, Department of Gynecology and Obstetrics, Istanbul Aydin University, Istanbul, Turkey; 5 Yasam Kemal Akpak, Department of Gynecology and Obstetrics, Sudan Nyala-Turkey Education and Research Hospital, Sudan

**Keywords:** Pregnancy, Body mass index, Weight gain, Obesity, Nutrition

## Abstract

**Objective:**

To investigate the adaptation of pregnant women to the recommended weight gain range according to body mass index (BMI) and to determine the factors affecting them.

**Methods:**

This cross-sectional study was performed in a university hospital’s obstetrics and gynecology unit (tertiary center) in Turkey. This study was conducted between March 2018 and August 2018 (6 months) in pregnant women. Pregnant women with chronic disease and receiving treatment during antenatal follow-up, with twin pregnancy, with a fetus with a congenital abnormality, and nutritional disturbance were excluded from the study. Eight hundred twelve pregnant women with normal antenatal follow-up and who volunteered to participate were included in the study.

**Results:**

The mean age of the participants was 27.66 ± 5.05 years. The mean weight and BMI before pregnancy were near standard in all participants. The group with the highest rate of recommended weight gain according to BMI before pregnancy was the group with low weight pregnant women. The ideal weight gain rate in all groups was 32%.

**Conclusions:**

The groups with overweight and obese pregnant women according to BMI before pregnancy had the highest rates of weight gain, above the recommended limits. BMI before pregnancy directly affects weight gain during pregnancy and the importance of pre-pregnancy counseling and weight loss is emphasized once again.

## INTRODUCTION

The nutritional needs of mother candidates increase in line with the physiologic changes that occur during pregnancy and the need for fetal growth. During pregnancy and/or breastfeeding, many women have some prejudices and misconceptions about eating everything and the intake of certain food types.^1^ Adequate weight gain during pregnancy is important for pregnancy, delivery, and the long-term health consequences of the mother and child. Excessive weight gain during this period was found to be related with gestational diabetes, hypertension, preeclampsia, vaginal birth assisted with intervention, cesarean section and early birth.^2^-^4^ In addition to its maternal effects, excessive weight gain during pregnancy includes many metabolic risks, especially macrosomia, and health problems in childhood and adulthood in the newborn.^5^ Inadequate weight gain increases the risk of having low-birth-weight infants, premature infants, and neonatal diseases.^6^,^7^

There is no clear worldwide consensus on ideal weight gain. The guidelines of the Institute of Medicine (IOM), which were established in 1990 and revised in 2009, are the most widely used guidelines in the world, although initially only used in the United States. The IOM gave weight recommendations that should be taken during pregnancy according to maternal body mass index (BMI) before pregnancy. According to this guide, healthy women with low weight (BMI: less than 18.5 kg/m^2^) should gain 12.5-18 kg, normal weight women (BMI: 18.5-24.9 kg/m^2^) 11.5-16 kg, overweight women (BMI: 25-29.9 kg/m^2^) 11-14 kg, and obese women (BMI greater than 30 kg/m^2^) should only gain 5-9 kg during pregnancy. The IOM guide states that in prenatal health care, the BMI of every pregnant woman should be calculated in the first prenatal follow-up, the appropriate weight gain according to BMI should be recommended, and nutritional and physical counseling should be performed.^8^ In some studies, it was found that pregnant women who were recommended weight gain according to IOM guidelines had a higher probability of gaining appropriate weight than those who were not given recommendations.^9^

In this study, we aimed to investigate the relationship between BMI before pregnancy and gestational weight gain in Turkish women, and to compare their weight gain during pregnancy with the weight gain recommendations of the IOM.

## METHODS

Our research was planned as a cross-sectional study. Pregnant women with full and regular medical records who were in the 38th or above gestational week and were admitted to a University Hospital’s obstetrics and gynecology unit between March 2018 and August 2018 (six months) were included. All antenatal follow-ups of pregnant women were performed at this outpatient clinic. Pregnant women with chronic disease and receiving treatment during antenatal follow-up, with twin pregnancy, with a fetus with a congenital abnormality, and those with nutritional disturbance were excluded. Eight hundred twelve pregnant women with normal antenatal follow-up and who volunteered to participate were included.

The data were obtained using a data collection Form prepared by the researchers. The data collection Form consisted of 13 questions that questioned sociodemographic characteristics, obstetric characteristics, and BMI. The BMIs of the pregnant women were calculated with height and weight measurements in the initiation of pregnancy obtained from prenatal follow-up cards. The data collection Form was completed during face-to-face interviews. The weight gain rates of pregnant women and the eligibility according to the 2009 IOM weight gain guide were calculated.^8^ Written permission was obtained for this research from the Kocaeli University Ethics Committee for Non-interventional Clinical Research (KOU KAEK 01.03.2018, 2018/97), and from the institution where the data were collected.

### Statistical analysis

Data were analyzed using IBM SPSS V23. Compliance with normal distribution was studied using the Shapiro-Wilk test. The Chi-square test was used to compare categorical data. Data without normal distribution are presented in the form of median (minimum-maximum) values. Categorical data are presented as frequency (percentage) values. The level of significance was accepted as P < 0.05.

## RESULTS

The sociodemographic and obstetric characteristics of 812 pregnant women included in this study are shown in **Table-I**. The mean age of pregnant women was 27.66 ± 5.05 (minimum:18; maximum: 47) years. Around one-quarter (26.5%) of the participants were primary school graduates and 20.9% were university graduates. When examined in terms of economic situation, the ratio of those whose incomes were equal to their expenditure was 85.1%. The mean number of pregnancies was 2.30 ± 1.41 (minimum: 1; maximum: 8), parity was 0.98 ± 1.04 (minimum: 0; maximum: 5), and gestational week was 38.97 ± 1.13 (minimum: 34; maximum: 44). The mean BMI of the pregnant women was 24.84 ± 4.62 (minimum: 13.63; maximum: 40.0) kg/m^2^, and the mean weight before pregnancy was 63.74 ± 11.93 (minimum: 38; maximum: 104) kg. According to the BMI values, 3.2% of pregnant women were low weight, 52.6% were normal weight, 27.5% were overweight, and 16.7% were obese (**Table-I**).

**Table I:** Sociodemographic features of the pregnant women.

Sociodemographic features	n	%
***Educational status***		
Illiterate	42	5.2
Primary school	215	26.5
Secondary school	165	20.3
High school	220	27.1
University	170	20.9
***Marital status***		
Married	804	99
Single	6	0.7
Divorced	2	0.2
Economic status		
Income less than expense	82	10.1
Income equal to expense	691	85.1
Income more than expense	39	4.8

*Obstetrics features*	*Mean-Standard* Deviation	*Minimum-maximum*

Gravida	2.30 ± 1.41	1-8
Parity	0.98 ± 1.04	0-5
Abortion	0.30 ± 0.67	0-6
Live birth	0.90 ± 1.01	0-5
Gestational week	38.97 ± 1.13	34-44

	*Mean- Standard* Deviation	*Minimum-maximum*

Weight before pregnancy	63.74 ± 11.93	38-104
Total weight taken during pregnancy	14.42 ± 5.53	5 - 39
Body mass index before pregnancy	24.84 ± 4.62	13.63-40.0

Body mass index classification (kg/m^2^)	*n*	*%*

Low weight < 18.5	26	3.2
Normal weight 18.5-24.9	427	52.6
Overweight 25.0-29.9	233	27.5
Obese ≥ 30.0	136	16.7

The results of the comparison of total weight gain and recommended weight gain according to BMI in pregnancy are shown in Table-II. There was a statistically significant difference between total weight gain and recommended weight gain according to BMI (p < 0.001). Some 65.4% of those with low weight, 34.4% of normal weight, 24.2% of those who were overweight, and 33.8% with obesity gained weight within the recommended limits. Weight gain less than the recommended limits was found in 26.9% of those with low weight, 24.1% of normal weight women, 8.5% of those who were overweight, and 1.5% of women with obesity. Some 7.7% of those with low weight, 41.5% of normal weight women, 67.3% of those who were overweight, and 64.7% of women with obesity gained more weight than the recommended limits (p < 0.001).

**Table II:** Comparison of total weight gain and recommended weight gain according to BMI in pregnancy and the accordance between them.

		Total weight gain in pregnancy				

BMI Classification	Recommended weight gain according to body mass index by the IOM guideline **	Pregnant women with weight gain in the recommended limits (n/%)	Pregnant women with less weight gain than the recommended limits (n/%)	Pregnant women with more weight gain than the recommended limits (n/%)	P	χ^2^
Low weight	12.5-18.0 kg	17 (65.4)*	7 (26.9)	2 (7.7)*	< 0.001	92.33
Normal weight	11.5-16.0 kg	147 (34.4)	103 (24.1)*	177 (41.5)*		
Overweight	7.0-11.5 kg	54 (24.2)*	19 (8.5)*	150 (67.3)*		
Obese	5.0-9.0 kg	46 (33.8)	2 (1.5)*	88 (64.7)*		

BMI= Body mass index; SD= Standart deviation; min-max= minimum-maximum.

* It shows the cells from which the difference originates;

** IOM (Institute of Medicine) 2009 Weight Gain During Pregnancy: Reexamining the Guidelines (metric equivalent of the values which were given as pounds (lbs) in the original).

**Graph.1:**
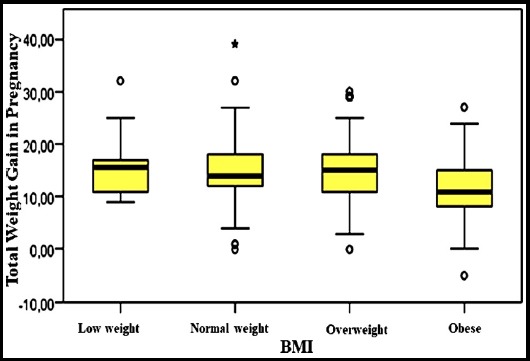
The comparison between total weight gain in pregnancy and BMI before pregnancy.

## DISCUSSION

Our study group consisted of pregnant women, 48% of whom had an education level of high school or above, 85% of whom had an average socioeconomic level (i.e. 85% were middle class), whose mean age was 27.66 ± 5.05 years, which means a young population with near-normal mean weight and BMI before pregnancy. The group with the highest rate of weight gain in line with the recommended weight gain was the low weight pregnant women. The ideal weight gain rate in all groups was 32%. The groups with overweight and obese patients according to BMI before pregnancy had the highest rates of weight gain, above the recommended amount of weight gain. BMI before pregnancy directly affected weight gain during pregnancy and the importance of pre-pregnancy counseling and weight loss was emphasized once again.

Thirty to 70% of women gain excessive weight during pregnancy and after each pregnancy they gain an average of 2 kg to 5 kg of extra weight.^10^,^11^ Long-term follow-up studies of pregnant women who gained more weight than they should showed that the risk of obesity was increased by 300% in the long-term.^10^ In a study of 1617 patients, being overweight and obese before pregnancy increased the risk of having macrosomic or large-for-gestational-age (LGA) fetuses. In addition, being normal weight before pregnancy but gaining weight more than the IOM recommendations during pregnancy increased the risk of having macrosomic or LGA fetuses.^12^ This situation creates both a serious medical problem and an economic dilemma.^10^ The prevalence of pregnant women gaining more weight than the recommended limits according to BMI before pregnancy is high and is still increasing. In a large-scale study conducted in Canada, a country with a high socioeconomic level, this rate was found as 59.4%.^13^ The rate of pregnant women who gained more weight than the recommended limits according to BMI before pregnancy was found as 51.3% in our study. This finding was in line with the results of a current review and meta-analysis in which this rate was reported as 51% in the United States, 51% in Western Europe, and 37% in Eastern Asia. The rate of pregnant women with higher BMI before pregnancy and the rate of pregnant women who gained more weight than the recommended limits were higher in the United States and Western Europe than in Eastern Asia. However, if regional BMI classifications are used in Eastern Asia, then the rate of excess gestational weight gain (GWG) is similar across the three continents.^14^

Pregnant women who gain less weight than the recommended limits are normal or low weight women, especially in the Far East and Asia.^4^,^6^ We also found that normal and low weight pregnant women had the highest rate of low GWG than the recommended limits. Weight gain in women is associated with ethnicity and culture, especially during pregnancy, and many studies have shown this. A study performed in Australia showed that the rate of early and excess GWG was highest in overweight and obese pregnant women who also had low socioeconomic levels.^15^ In addition, it was observed that pregnant women belonging to this low socioeconomic group did not know or were prone to ignore that their current weight was high and that their excess weight negatively affected their newborns.^15^ Our population had an average socioeconomic level, but the general economic situation and social structure of each country should be evaluated within itself. However, this socioeconomic situation and parity should be taken into consideration when selecting a target group in the pregnant population because primiparous women are prone to gaining more weight than they should in pregnancy compared with multiparous women.^14^

In a prospective study performed in Algeria, pregnant women with more GWG than the recommended limits were overweight or obese according to BMI in pregnancy.^16^ In another prospective study conducted in Australia, the data of pregnant women who were followed up beginning from the 20 th gestational week were evaluated and those with BMI less than 25 kg/m^2^ were classified as healthy and women with BMI greater than 25 kg/m^2^ were classified as overweight. The odds ratio of healthy pregnant women to gain more weight than the recommended limits was 0.6 (95% Confidence Interval: 0.4 to 0.8) and 1.4 (95% CI: 1.1 to 2.0) in overweight pregnant women.^17^ Although in our study, the groups with overweight and obese pregnant women according to BMI before pregnancy had the highest rates of weight gain above the recommended limits, it was observed that this effect continued during pregnancy. In addition to giving education and losing weight before pregnancy, the importance of personalized diet, exercise, and nutrition education during pregnancy is clear. Implementation of diet, exercise, and training at the same time is important. In a randomized controlled study in England, 1.555 pregnant women with a BMI > 30 kg/m^2^ who were randomly selected between 15-19 gestational weeks, conceived of a broad socioeconomic and racial spectrum were included in the study. Behavioral training was given to the study group once per week and only antenatal follow-up was performed in the control group. However, a behavioral intervention that addressed diet and physical activity did not improve obstetric and perinatal outcomes, did not prevent development of gestational diabetes, and did not reduce the frequency of LGA or macrosomic babies.^18^ In another study, duration of stay in hospital, frequency of diseases such as respiratory distress syndrome (RDS) and macrosomia in the newborns of pregnant women with BMI > 25 kg/m^2^ were evaluated. Statistically significant results were obtained in all parameters in the diet group in which behavioral training was added.^19^ In randomized controlled studies, by adding diet and exercise to behavioral interventions before pregnancy, BMI was reduced in 12-month follow-up and it was shown that decreasing BMI by increasing physical activity positively affected the prognosis of pregnancy.^20^,^21^

In a review that investigated factors other than BMI before pregnancy that affected GWG, it was found that GWG was not associated with negative emotions such as depression, anxiety and stress, but negative body image of the pregnant woman, inability to enjoy her own body, and her attitude towards weight gain were associated with excessive GWG.^22^

Pregnancy is the most appropriate period to investigate this international problem and to find a solution.^23^ It has been shown that patients want to receive information from their healthcare workers and they trust them.^24^ In addition, it seems effective to take advantage of technology. In a study, it was observed that pregnant women who were delivered warnings about GWG, beginning from the 16th gestational week by telephone, webinar, SMS, e-mail or application gained statistically less weight.^25^

### Limitations of the study

This study was conducted in a single university hospital and in a relatively small sample group. More effective results could be obtained if a prospective cohort study was performed with a larger number of pregnant women. Although the weight of the patients was measured by a single, fixed and accurate weightier, the height of the patients recorded according to their own statements. These statements can cause problems even if they correctly measured their height. More demographic data and emotional and behavioral parameters could be added to the study design.

## CONCLUSION

Excessive weight is a major and neglected public health burden. Women in reproductive age are both at high risk for complications and are also target groups in terms for interventions. Ethnicity and BMI affect the range of weight that should be gained during pregnancy. The efficacy of individualized behavioral training, diet, and exercise is still not clear but should be investigated. It is thought that those who are overweight and obese before pregnancy should be brought to the ideal weight and lifestyle with a complex intervention, and those who are low weight should be protected by state policy and reach the ideal care conditions, which will improve antenatal and postnatal outcomes.

### Authors’ Contribution

**RO, YAA, SDA, BAS & YKA:** Conceived, designed and did statistical analysis & editing of manuscript.

**RO & SDA:** Did data collection and manuscript writing.

**YAA, BAS & YKA:** Did review and final approval of manuscript..
